# Pulvinar subdivisions and connectivity patterns across primate species: a comparative perspective

**DOI:** 10.3389/fnana.2026.1794659

**Published:** 2026-04-28

**Authors:** Juliana G. M. Soares, Amaro R. A. Correia, Ricardo Gattass, Bruss Lima

**Affiliations:** 1 Laboratory of Cognitive Physiology, Instituto de Biofísica Carlos Chagas Filho, Universidade Federal do Rio de Janeiro, Rio de Janeiro, Brazil; 2 Programa de Pós-Graduação em Ciências Cirúrgicas, Faculdade de Medicina, UFRJ, Rio de Janeiro, Brazil

**Keywords:** capuchin monkey, connectivity, cortical areas, new world monkeys, primate, pulvinar, superior colliculus, thalamus

## Abstract

With the advances in our ability to perturb brain activity in recent years, new stimulation techniques have become essential tools in human neuroscience. Non-invasive stimulation methods, such as transcranial magnetic stimulation (TMS), as well as deep brain stimulation (DBS) delivered invasively to access deep brain structures, have been applied in both basic and clinical research and in the treatment of neurological conditions including Parkinson’s disease, essential tremor and epilepsy. In the context of epilepsy, neuromodulatory interventions have demonstrated encouraging results in reducing seizure frequency, bringing attention to the thalamic pulvinar nucleus as a potential target for stimulation in drug-resistant cases. To advance these and future therapies, it is necessary to have a more detailed understanding of the subdivisions and connectivity patterns of these nuclei. Although some human studies have employed diffusion imaging and fMRI, much of the current knowledge of pulvinar connectivity still comes from non-human primate (NHP) studies. The aim of this study is to review the cortico-pulvinar connectivity patterns of distinct pulvinar subregions across NHP species, alongside available human studies, to help optimize future basic and clinical research.

## 1 Introduction

The pulvinar is the largest thalamic nucleus in primates, occupying the posterior portion of the thalamus. During evolution, its development occurred concurrently with the expansion and differentiation of the temporo-parieto-occipital cortex, with which it is richly interconnected (Harting et al., 1972; Rakic, 1974). Using cytoarchitectural criteria, the pulvinar was subdivided into medial (PM), lateral (PL), and inferior (PI) portions (Walker, 1938). Later, other subdivisions were proposed based on connectivity, electrophysiological topographic mapping or chemoarchitecture. However, these subdivisions do not always match one another (Gattass et al., 2018).

The role of the pulvinar in visual function has been demonstrated by the existence of a retinotopic organization, as well as its topographic connections with visual cortical areas (Allman et al., 1972; Gattass et al., 1978a; Bender, 1981; Ungerleider et al., 1983). The multiple representations of the visual field in the inferior and lateral portions of the pulvinar and their structural and metabolic diversity have led researchers to consider the pulvinar as a complex of functional nuclei (Gattass et al., 1978a; Cusick et al., 1993; Soares et al., 2001).

Other studies, both in monkeys and humans, have suggested the involvement of the pulvinar in visual attention (Petersen et al., 1987; Rafal and Posner, 1987; LaBerge and Buchsbaum, 1990; Kastner et al., 2004; Zhou et al., 2016; Chen et al., 2023) and in synchronizing neural activity across cortical areas, effectively modulating the efficacy of cortico-cortical information transfer (Jones, 2001; Saalmann and Kastner, 2009; Saalmann et al., 2012; Fiebelkorn et al., 2019). Pulvinar inactivation resulted in a change in response and in contrast gain of neurons in visual areas V1, V2, and V4 (Soares et al., 2004; Purushothaman et al., 2012), reinforcing the role of the pulvinar in the modulation of cortical neuronal activity.

The dorsal pulvinar, in turn, located above the brachium of the superior colliculus (SC), including PM and the dorsal portion of PL (PL_*D*_), has strong connections with frontoparietal cortical areas and have been involved in the execution of saccades and other visuomotor behaviors such as the selection and the execution of reaching and grasping movements (Wilke et al., 2010; Dominguez-Vargas et al., 2017). PM and PL_*D*_ are also involved in emotional face recognition and in language processing (Assaf et al., 2006; Ward et al., 2007; Crosson, 2013; Meaux and Vuilleumier, 2016).

Medial pulvinar has also been implicated as a selective hub critical for lexical access in naming. Naming an object requires dynamic coordination of a broad network of brain regions. Damage to any one of these regions can result in an inability to accurately retrieve the name of an object, an anomic aphasia. Direct pulvinar stimulation induced anomia, suggesting that this region may be a critical causal node or densely connected with the naming network (Woolnough et al., 2026).

The pulvinar is also densely connected to cortical areas involved in multisensory integration. It has been proposed that the pulvinar combines multiple sources of sensory information to enhance fast responses to the environment, while also playing the role of a general regulation hub for adaptive and flexible cognition (Froesel et al., 2021). As we describe in more detail in see section “5 Clinical implications,” the pulvinar complex is implicated in neurodegenerative disorders such as Alzheimer’s and Parkinson’s disease (PD) and various other disorders such as schizophrenia, spatial neglect, attention deficit disorder, autism spectrum disorders and epilepsy (Byne et al., 2001; Arend et al., 2008; Barron et al., 2014; Kleinhans et al., 2011; Capecchi et al., 2020; Velioglu et al., 2023). Pulvinar subregions are serving as therapeutic targets and abnormalities in these regions can function as biomarkers for some of these disorders. For example, Responsive Neurostimulation (RNS) in the pulvinar nuclei in individuals with bilateral multifocal posteriorly dominant drug-resistant epilepsy results in significant reduction in disabling seizures (Wang R. et al., 2024). Here, we review the organization of the pulvinar complex and the cortico-pulvinar connectivity patterns of distinct pulvinar subregions of New and Old World monkeys and include more recent evidence from human studies to help optimize future basic and clinical studies.

## 2 Pulvinar subdivisions across primates

**FIGURE 1:**
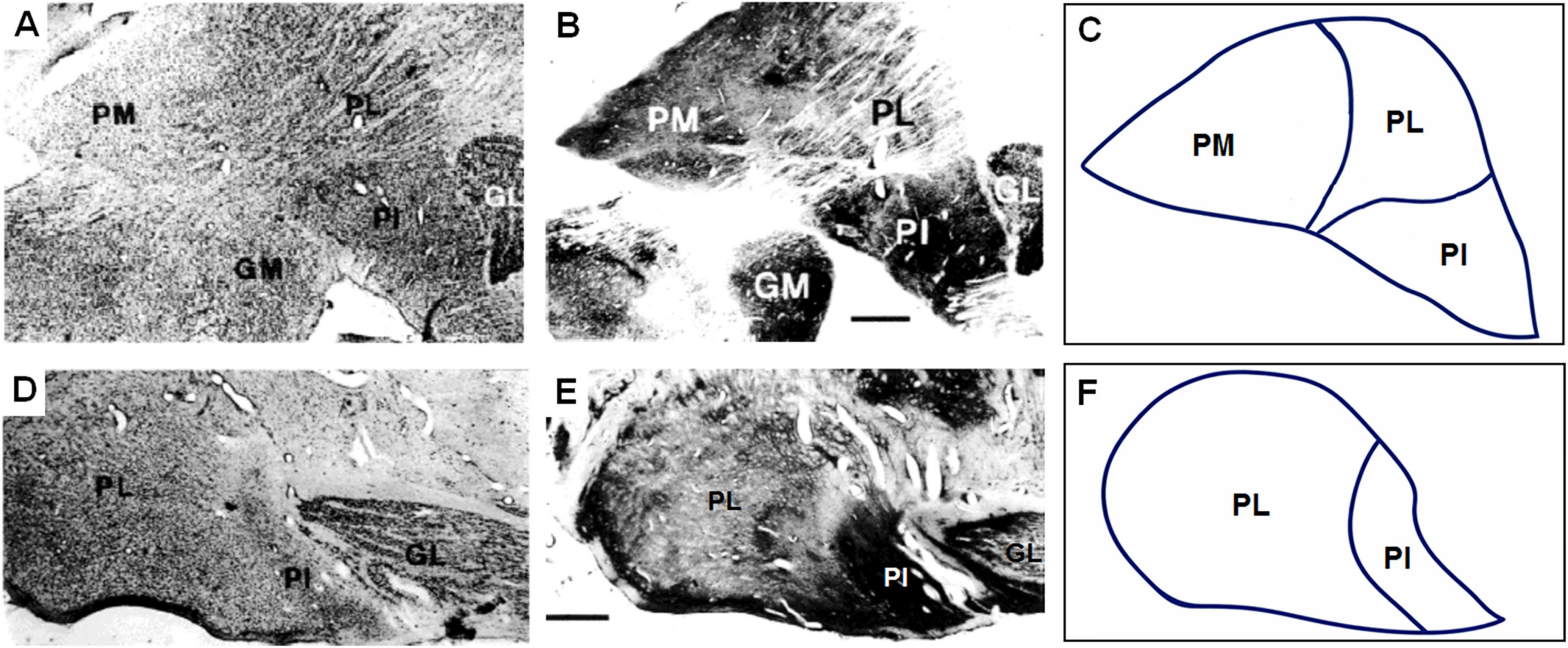
Cytoarchitectonic subdivisions of the pulvinar according to Walker (1938). Photomicrographs of coronal **(A,B)** and parasagittal **(D,E)** sections of the capuchin monkey pulvinar stained with Nissl **(A,D)** and cytochrome oxidase **(B,E)** methods. **(C,F)** Outline drawings of coronal and parasagittal sections illustrated in panels **(A,D)**, respectively, showing the cytoarchitectonic subdivisions of the pulvinar. PM, medial pulvinar; PL, lateral pulvinar; PI, inferior pulvinar; GL, lateral geniculate nucleus; GM, medial geniculate nucleus. Scale bar = 1 mm. [Modified from Soares et al. (2001)].

Walker (1938) subdivided the pulvinar of the macaque monkey into three portions based on topography and cytoarchitecture: the medial pulvinar (PM), the lateral pulvinar (PL), and the inferior pulvinar (PI). Olszewski (1952) added an anterior subdivision named pulvinar oralis (PO), between the centromedian (CM) and ventral posterior lateral (VPL) thalamic nuclei. In New World monkeys, such as the squirrel (*Saimiri sciureus*), the marmoset (*Callithrix jacchus*) and the capuchin (*Sapajus apella*, formerly *Cebus apella*) monkeys, we observe a similar subdivision of the pulvinar (Mathers, 1972; Spatz and Erdmann, 1974; Soares et al., 2001). Figure 1 shows these subdivisions of the pulvinar in the capuchin monkey.

**FIGURE 2:**
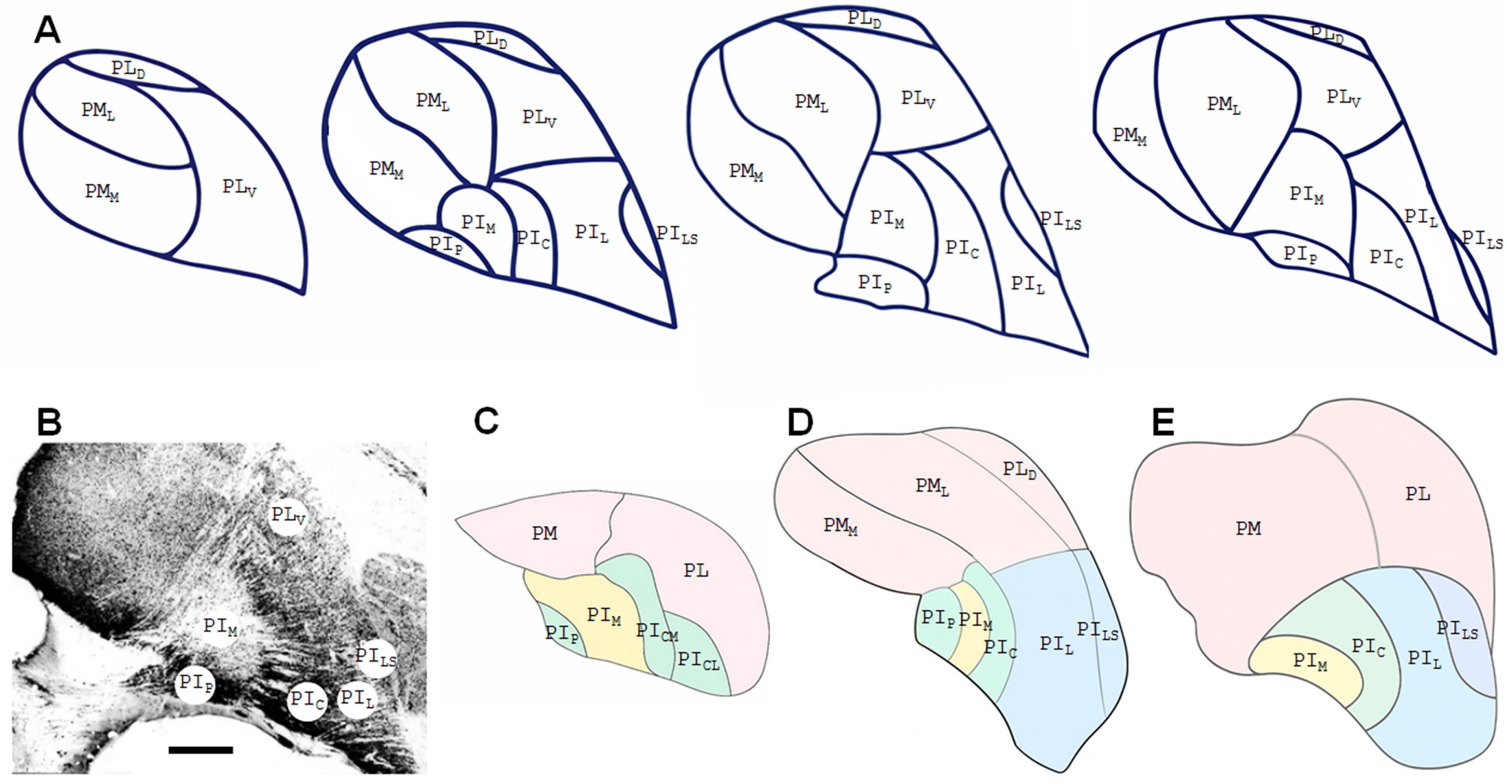
Chemoarchitectural subdivisions of the pulvinar in the capuchin **(A,B)**, marmoset **(C)** and macaque **(D)** monkeys and in the human **(E)**. **(A)** Outline drawings of coronal sections of the capuchin monkey pulvinar spaced 400 μm apart showing the subdivisions based on calbindin immunostaining. **(B)** Photomicrograph of a coronal section of the capuchin monkey pulvinar reacted for calbindin [modified from Soares et al. (2001)]. Note that PI can be subdivided into PI_*P*_ (posterior), PI_*M*_ (medial), PI_*C*_ (central), PI_*L*_ (lateral), and PI_*LS*_ (lateral shell) regions, PL can be subdivided into ventral (PL_*V*_) and dorsal (PL_*D*_) portions and PM can be subdivided into medial (PM_*M*_) and lateral (PM_*L*_) portions. **(C)** Outline drawing of a coronal section of the marmoset pulvinar showing subdivisions based on Kaas and Lyon (2007) where the inferior pulvinar contains four divisions: posterior (PIp), medial (PIm), central medial (PIcm), and central lateral (PIcl). **(D)** Outline drawing of a coronal section of the macaque pulvinar showing the subdivisions proposed by Cusick et al. (1993), Gutierrez et al. (2000). **(E)** Outline drawing of a coronal section of the human pulvinar showing the subdivisions based on reports by Cola et al. (1999). Scale bar = 1 mm.

Lin and Kaas (1979), based on architectonic characteristics, distinguished three nuclei in PI of the owl monkey: the medial nucleus, IPm, the central, IPc, and the posterior nucleus, IPp. IPc occupies about 70% of PI, while IPm occupies about 20% of PI and extends dorsally across the brachium of the SC. Following this pattern and based on the chemoarchitecture of the pulvinar, studies in squirrel and macaque monkeys revealed five subdivisions of PI that include, in addition to PI, ventral portions of PL and PM. PI_*M*_, a calbindin-poor medial zone, separate the posterior (PI_*P*_) and the central (PI_*C*_) regions, intensely stained for calbindin. PI_*L*_, lightly stained for calbindin, and the most lateral shell zone (PI_*Ls*_) occupies the ventral portion of PL (Cusick et al., 1993; Gutierrez et al., 1995). Stepniewska and Kaas (1997) proposed yet a subdivision of PI_*C*_ in the lateral (PI_*CL*_) and medial (PI_*CM*_) portions. PL and PM were also subdivided based on chemoarchitecture in ventral (PL_*V*_) and dorsal (PL_*D*_) portions, and lateral (PM_*L*_) and medial (PM_*M*_) portions, respectively (Gutierrez et al., 2000). These chemoarchitectonic subdivisions are preserved to a certain degree across species of New and Old World monkeys, despite some differences in terminology. The subdivisions of the pulvinar in the capuchin monkey based on chemoarchitecture are illustrated in Figures 2A,B. Figure 2 also illustrates outline drawings of coronal sections showing the subdivisions of the marmoset (Figure 2C), macaque monkey (Figure 2D) and of the human (Figure 2E) pulvinar.

**FIGURE 3:**
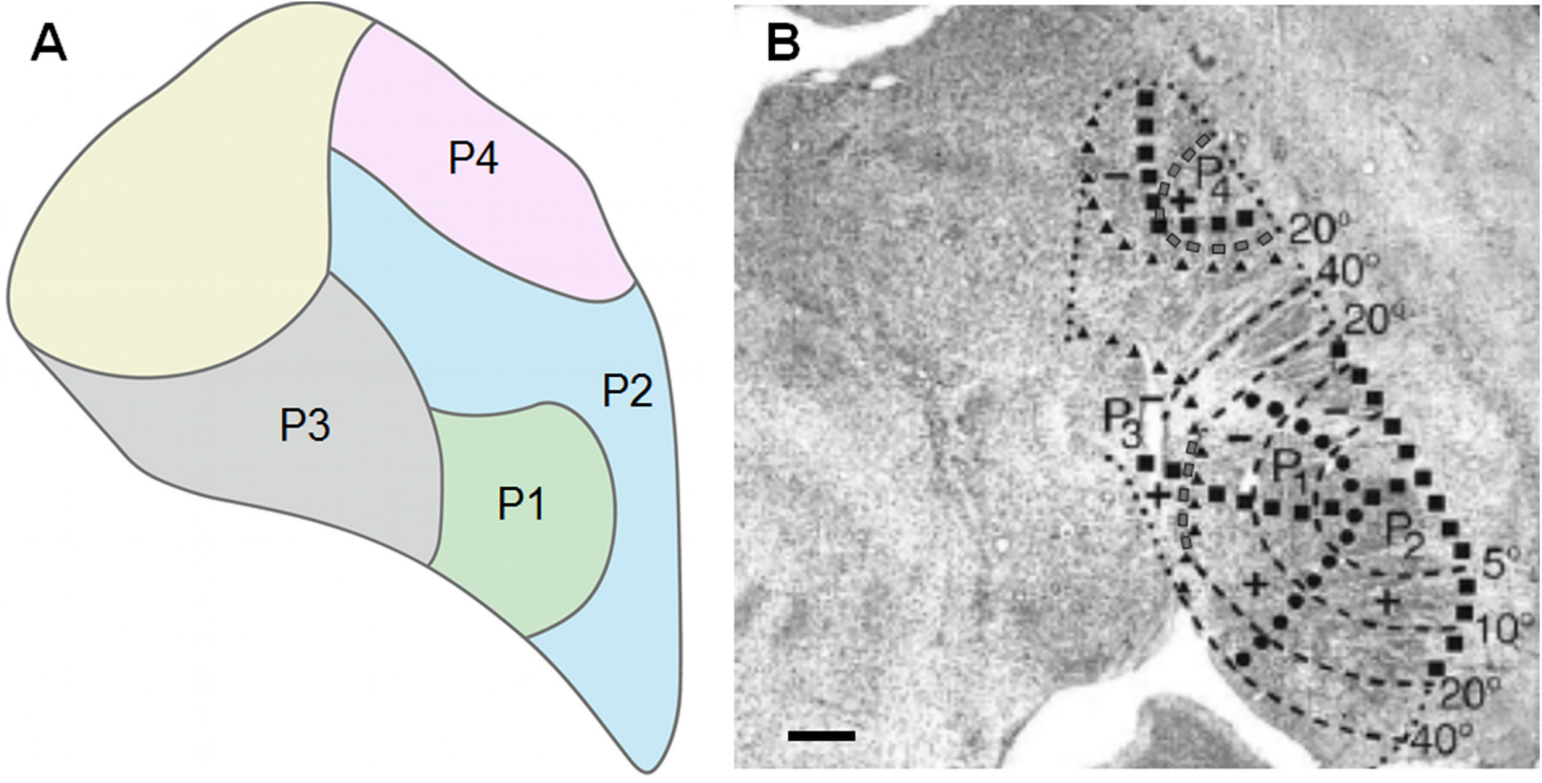
Visual topography of the pulvinar projection fields (P1, P2, P3, and P4) of the macaque pulvinar revealed after tracer injections in cortical areas V1, V2, V4, PO, and MT **(A)**, based on Ungerleider et al. (2014). **(B)** The visual maps are shown superimposed on a coronal section stained for Nissl. Solid circles indicate the representation of the vertical meridian, solid squares indicate the representation of the horizontal meridian, heavy dashes indicate isoeccentricity lines, gray colored dashes indicate isoeccentricity lines in areas of coarse topography, small solid triangles indicate the borders of P3 and P4, and small dotted lines indicate the borders of the pulvinar fields. The plus sign indicates the upper visual field representation, and the minus sign indicates the lower visual field representation. Scale bars = 1 mm. [Modified from Ungerleider et al. (2014)].

Studies of the pulvinar organization based on electrophysiological recordings revealed topographic maps in PI and PL of New and Old World monkeys (Allman et al., 1972; Gattass et al., 1978a; Bender, 1981; Petersen et al., 1985). Analysis of the electrophysiological data associated with connectivity studies (Standage and Benevento, 1983; Ungerleider et al., 1983, 1984, 2014; Adams et al., 2000; Soares et al., 2001; Gattass et al., 2014) revealed three visuotopic fields in the pulvinar, namely P1, P2, and P4 and one field named P3 that does not seem to have a well-defined visuotopic map (see Figure 3 for data obtained in the macaque monkey). The P1 field, as described by Ungerleider et al. (1983), includes PI_*CL*_ and the ventromedial portion of PL (PL_*VM*_). The P2 field corresponds to the ventrolateral portion of PL (PL_*VL*_). The peripheral visual field of P1 is represented anteriorly in the medial portion of PI, while the central visual field is represented more posteriorly in the medial portion of PL. The vertical meridian is represented on the lateral edge, while the horizontal meridian is represented obliquely from lateral to medial across the nucleus. The upper field is represented ventrally, while the lower field is represented dorsally. P1 and P2 share the representation of the vertical meridian, while P2’s horizontal meridian representation is a continuation of P1’s, in a way that the foveal region is represented at the lateral border of the pulvinar. P4 is located in dorsal portion of PL (PL_*D*_), adjacent to the lower field representation of P2. The representation of the vertical meridian is located on the dorsal edge, while the representation of the horizontal meridian exits the dorsal edge so that the upper visual field occupies the dorsal and anterior portions of P4, while the lower visual field is located ventral and posterior. P4 (PL_*D*_) would be equivalent to Pdm described by Petersen et al. (1985). P3 is located posteromedially in PI but also includes small adjacent portions of PL and PM. P3 borders were estimated based on its connectivity with middle temporal visual area (MT) and would encompass the PI_*P*_, PI_*M*_ and PI_*CM*_ subdivisions (Gattass et al., 2014).

In addition, Liu et al. (2021) performed a series of small injections of retrograde tracers in area V2 of macaques and identified three additional retinotopic maps in PL/PI_*CL*_, leading to a total of five maps that they named Clusters 1–5. They suggested that P1 should be further subdivided into two maps and that there are two more maps: one between P1 and P2, and another dorsal to them, whereas the topography of Cluster 5 would be consistent with that of the previously suggested P2.

## 3 Pulvinar connections in New and Old World monkeys

**FIGURE 4:**
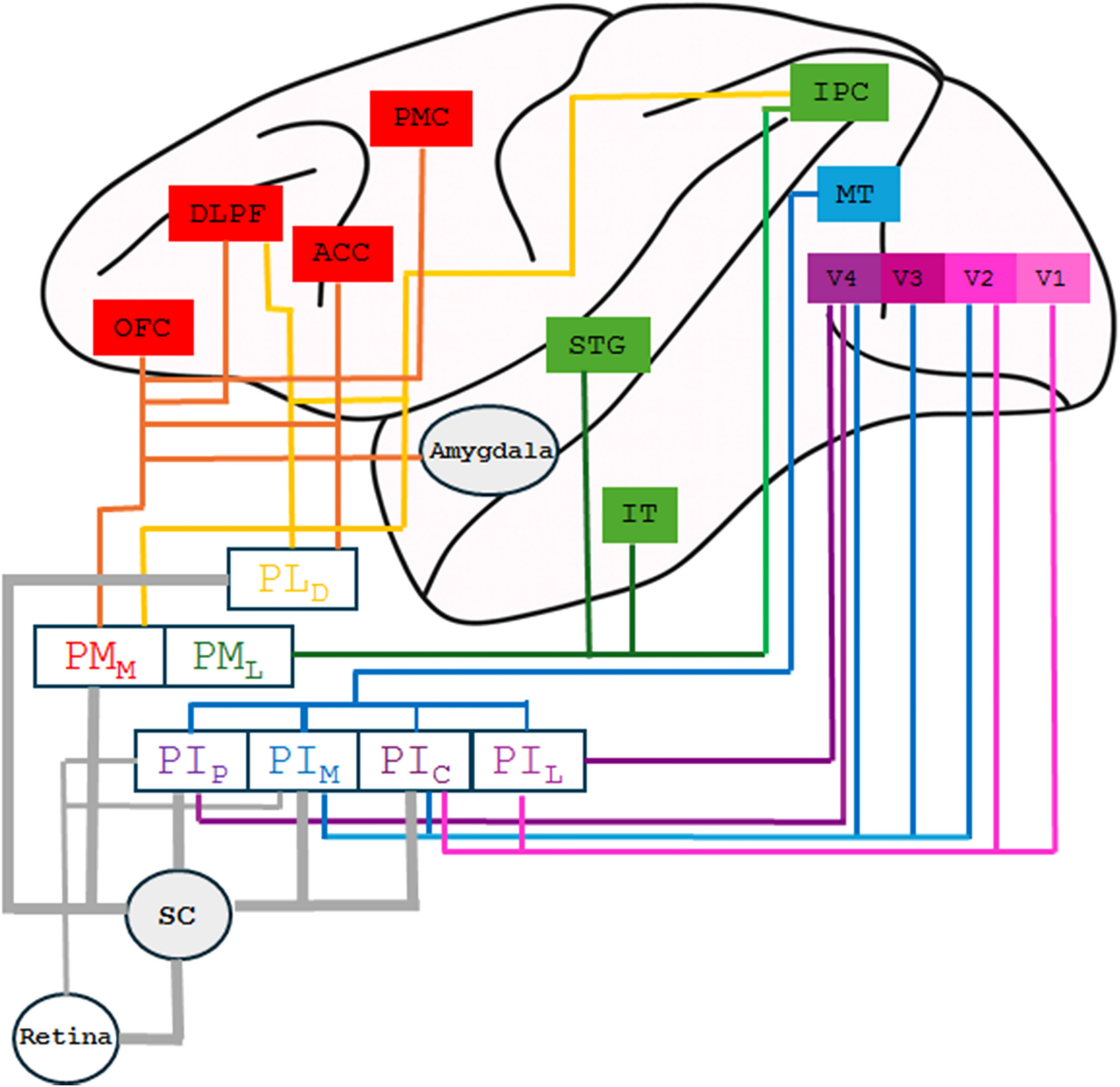
Diagram of the anatomical connections of the pulvinar subdivisions. Rectangles representing cortical regions are depicted on the brain hemisphere outline drawing, whereas subcortical structures are represented by rectangles and circles on the inferior portion of the figure. Colored lines illustrate bidirectional anatomical connections. Projections originating in the retina and superior colliculus (SC) (gray lines) are unidirectional. PI_*P*_, PI_*M*_, PI_*C*_, and PI_*L*_: posterior, medial, central, and lateral subdivisions of the inferior pulvinar, respectively. PM_*M*_, PM_*L*_: medial and lateral subdivisions of the medial pulvinar, respectively. PL_*D*_, dorsal subdivision of the lateral pulvinar; ACC, anterior cingulate cortex; DLPF, dorsolateral prefrontal cortex; IPC, inferior parietal cortex; IT, inferotemporal cortex; OFC, orbitofrontal cortex; PMC, premotor cortex; STG, superior temporal gyrus.

The pulvinar nucleus receives inputs mainly from the SC and the retina and has reciprocal connections with most cortical regions. These connections seem to have a gradient which reflects cortical topography. Namely, posterior and lateral parts of the pulvinar are more likely to be connected to the posterior regions of the cortex, while the anterior and medial pulvinar are more likely to be connected to anterior cortical regions. The pulvinar connections also exhibit a dorso-ventral gradient, where the ventral pulvinar, including PI and PLv, is connected mainly to occipital and temporal cortices, while the dorsal pulvinar, consisting of PM and PL_*D*_, is connected to frontal, parietal, and cingulate cortices (Shipp, 2003). In Figure 4, we present a summary of the pulvinar connections based on neuroanatomical tracing studies in both New and Old World monkeys (Adams et al., 2000; Gutierrez et al., 2000; Soares et al., 2001; Ungerleider et al., 2014; Gattass et al., 2014; Bridge et al., 2016; Bourne and Morrone, 2017).

### 3.1 Pulvinar connectivity with the retina and superior colliculus

Studies in macaque monkeys and baboons indicate a weak direct retinal projection to PI. They were found preferentially within the medial subdivision of PI, with some involvement of the posterior and central subdivisions (Campos-Ortega et al., 1970; Cowey et al., 1994; O’Brien et al., 2001). Warner et al. (2010) showed direct retinal projections to MT relay cells in PI_*M*_ of macaque while Kwan et al. (2019) identified contralateral retinal terminations primarily in PI_*M*_, with a few scattered inputs in PI_*CM*_ and PI_*CL*_ in the marmoset monkey.

Superior colliculus projections to the pulvinar were found in PM_*L*_, PM_*M*_, medial portions of PL_*D*_ and PL_*V*_ and in the ventromedial and in the dorsolateral portions of PI (Mathers, 1972; Benevento and Fallon, 1975; Trojanowski and Jacobson, 1975; Lin and Kaas, 1979; Benevento and Standage, 1983; Elorette et al., 2018). In the PI complex of owl monkeys, both IPp and IPc receive projections from the superficial layers of the SC, but the terminations in IPp are denser than those in IPc (Lin and Kaas, 1979).

Partlow et al. (1977) showed topographically organized SC projections to most of the macaque PI, with the lower visual field being represented dorsomedially, and the upper field ventrolaterally. The peripheral representation was located along the medial border, and the fovea representation at the dorsolateral angle, adjacent to the lateral geniculate nucleus (LGN). Benevento and Standage (1983), in addition to the projections to PI, described projections from SC to three zones in PL, one along the lateral border of caudal portion of PL, a second projection to rostral lateral portion adjacent to PI, and the last one located more medially in PL.

The identification of a subset of relay neurons in the macaque that both receive SC input and project to MT demonstrate a continuous functional path from SC to MT through the pulvinar in primates. Some of the relay neurons resided in two PI subdivisions, PI_*M*_ and PI_*P*_, as defined by immunostaining (Lyon et al., 2010; Berman and Wurtz, 2010, 2011). The pathway that connects the superficial layers of the SC to the dorsal visual cortices MT and V3 represents a second route from the retina to the visual cortex that bypasses the LGN. The fast transmission time estimated between the SC and MT suggests that this pathway may mediate motion detection, saliency processing, and saccadic suppression.

However, Kwan et al. (2019), using bidirectional tracer injections in the separate subdivisions of the PI, SC, retina and LGN in the marmoset monkey (*Callithrix jacchus*), demonstrated that the PI_*M*_ receives retinal but not SC inputs. The direct retinal projections to the pulvinar originate exclusively from widefield ganglion cells which overlap in morphology with SC-projecting widefield cells, yet do not appear to form collateral projections to the SC. On the other hand, PI_*P*_ and PI_*CM*_ receive both SC and LGN inputs.

Elorette et al. (2018) described projections from the SC that colocalized with cell bodies within the medial portions of PM, PO and PI, in the vicinity of the brachium of the SC, that were labeled by retrograde tracer injected into the lateral amygdala. These data could provide an anatomical substrate for the previously proposed pathway mediating the fast processing of emotionally salient information (Jones and Burton, 1976; LeDoux, 1996; Romanski et al., 1997; Stefanacci and Amaral, 2000).

### 3.2 Cortico-pulvinar connectivity

There are reciprocal and topographically organized projections from PI and PL to a large portion of the primate visual cortex, including the striate, prestriate, inferotemporal, and parietal cortices (Campos-Ortega and Hayhow, 1972; Ogren and Hendrickson, 1976, 1977; Benevento and Rezak, 1975, 1976; Rezak and Benevento, 1979; Gattass et al., 2018). The projections to area 18 are stronger than those to area 17 and are observed mainly in cortical layers IV, III, and I (Ogren and Hendrickson, 1977; Rezak and Benevento, 1979; Wong-Riley, 1977). Cortical projections to the pulvinar arise mainly from layer 5 and 6 pyramidal cells (Lund et al., 1975; Trojanowski and Jacobson, 1977; Levitt et al., 1995).

Studies of the V1-pulvinar connectivity showed two topographically organized regions in the pulvinar. Injection within the central cortical visuotopic representation resulted in the labeling of the lateral halves of PI and PL, while peripheral cortical injections showed more medial labeling. The vertical meridian was represented in the boundary between these two pulvinar regions (Campos-Ortega and Hayhow, 1972; Spatz and Erdmann, 1974; Ogren and Hendrickson, 1976, 1977). These projections zones are similar to regions described in previous electrophysiological recordings and later called P2 and P1, respectively (Allman et al., 1972; Bender, 1981, Gattass et al., 2018). Ungerleider et al. (2014), using multiple tracer injections in different eccentricities in V2 in macaque monkeys showed well-defined topographic maps in P1 and P2, and a cruder map in the dorsal portion of PL, called P4.

The projections from the pulvinar to MT originate mainly in the medial portion of PI and in a small portion of adjacent PM and PL (Lin et al., 1974; Standage and Benevento, 1983). The connections between MT and this region, later called P3, are reciprocal and topographically organized, with the lower visual field represented dorsally and the upper visual field represented ventrally. There is an expanded representation of central vision located caudally within this region, while peripheral vision is represented rostrally (Standage and Benevento, 1983; Soares et al., 2001). PI_*M*_, PI_*P*_, and PI_*C*_ also project to other dorsal visual stream areas such as medial superior temporal (MST) and fundus of the superior temporal sulcus (FST) (Baleydier and Morel, 1992; Kaas and Morel, 1993; Boussaoud et al., 1992). PL_*D*_ also has strong connections with MST, with the posterior parietal cortex, including lateral (LIP), ventral (VIP), medial (MIP), and anterior (AIP) intraparietal areas and with the superior parietal areas PE and PEa (Webster et al., 1993; Adams et al., 2000; Shipp, 2003).

Gattass et al. (2014) studied the pulvinar-V4 connectivity in the macaque monkey using anterograde and retrograde tracer injections in V4. Several clusters of labeled cells and terminals were found in P1, P2, P4 and mainly in the medial portion of P3 (Figure 3). The subdivisions of PI, PI_*P*_, PI_*M*_, and PI_*CM*_, as well as PL, also have connections with other ventral visual stream areas such as temporal areas FST and TEO (Boussaoud et al., 1992; Baleydier and Morel, 1992; Baizer et al., 1993; Webster et al., 1993; Adams et al., 2000; O’Brien et al., 2001; Warner et al., 2010).

Kaas and Lyon (2007) further proposed that the pulvinar nuclei could be segregated into two distinct groups based on their connectivity with the two cortical streams of visual information processing. More lateral nuclei of PI and the adjoining PL are mainly interconnected with areas of the ventral stream, largely devoted to object recognition and perception, while the medial nuclei of the PI complex are mainly interconnected with areas of the dorsal stream, specialized for visually guiding behavior and visuospatial processing (Mishkin and Ungerleider, 1982; Goodale and Milner, 1992). That way, the pulvinar would provide integration of information both within and between the two visual streams.

Inferior pulvinar and PM_*M*_ are also connected with the primary auditory cortex A1, the caudal and the rostral belts of the auditory cortex (cAC and rAC) and caudal superior temporal gyrus (STG) (Gutierrez et al., 2000; Kaas and Lyon, 2007).

The subdivisions of PM, PM_*M*_, and PM_*L*_, project to the temporal, parietal, insular and prefrontal areas. PM_*M*_ has connections with TE, MST, LIP, VIP, somatosensory, superior parietal and the inferior parietal areas (Baizer et al., 1993; Baleydier and Morel, 1992; Webster et al., 1993; Morel et al., 2005; Impieri et al., 2018; Homman-Ludiye et al., 2020). It also projects to the frontal eye field (FEF), dorsolateral and ventrolateral prefrontal orbito-frontal cortices and amygdala (Trojanowski and Jacobson, 1974; Romanski et al., 1997; Gutierrez et al., 2000). PM_*L*_ is interconnected with the visual areas V1, V2, V4, and TEO as well as with the intraparietal area LIP and STG (Benevento and Davis, 1977; Romanski et al., 1997; Shipp, 2003; Gutierrez et al., 2000). Both medial and anterior subdivisions of the pulvinar project to the premotor cortex and, to a lesser extent, to the supplementary motor area and the primary motor cortex (Morel et al., 2005; Córdoba-Claros et al., 2025).

Córdoba-Claros et al. (2025) demonstrated numerous neurons in PM that innervate, through branched axons, prefrontal and parietal or prefrontal and temporal areas. Other cells with different projection patterns are closely intermingled with them. These findings suggest that PM can function as a hub of brain-wide networks that support complex visual and social cognition, sensory-guided reaching, working memory and attention.

## 4 Functional organization and connectivity of the human pulvinar

The pulvinar, as measured by both volume and number of neurons, is proportionally much larger in humans than in monkeys or great apes (Armstrong, 1981). The PM, which projects to higher order association cortex, underwent a greater degree of expansion in human evolution than did the PI complex, which projects mainly to visual cortex. A study of the human PI complex (Cola et al., 1999), using histochemical techniques comparable to those used in monkeys, identified plausible homologs of most of the PI subdivisions (Figure 2E). Compared to monkeys, the human PI complex was observed to be proportionately smaller with respect to the pulvinar as a whole. It contains four histochemical zones, corresponding to the medial, central, lateral and lateral-shell (PI_*M*_, PI_*C*_, PI_*L*_, and PI_*LS*_) divisions of the PI complex in monkeys. Cola et al. (1999) could not identify in the human PI complex an area comparable to the PI_*P*_ of monkeys. They speculated that the PI_*P*_ could be an extension of PI_*C*_, rather than a distinct division of PI.

Studies using diffusion tensor imaging (DTI) tractography demonstrated that the human pulvinar is interconnected with subcortical structures as the SC, thalamus, amygdala and caudate nucleus, as well as with cortical visual areas V1, V2, V3, visual inferotemporal areas, posterior parietal association areas, primary motor cortex, frontal eye fields and prefrontal areas. Most of these results are consistent with the connectivity reported in monkey studies (Leh et al., 2008; Tamietto et al., 2012).

Tamietto et al. (2012), using DTI, found fiber connections between the pulvinar and the amygdala and also between the superior colliculus and the amygdala via the pulvinar. Reconstructed fibers connecting the pulvinar and the amygdala extended to/from the ipsilateral temporal pole, dorsal prefrontal cortex, orbitofrontal cortex, caudate, and SC. The amygdala appears to be connected to two different subregions of the ipsilateral pulvinar, namely its dorsomedial and its inferolateral portions. The dorsomedial portion includes multimodal neurons preferentially connected to the amygdala and to frontal areas and could be involved in conscious emotion perception. The inferolateral portions of the pulvinar, including ventromedial portions of PI and PL, are connected to visual areas and receive direct afferents from the SC and from the retina. This inferolateral subdivision of the pulvinar can be considered functionally homologous to the pulvinar regions recently found to send projections to the amygdala and is involved in the disynaptic SC-pulvinar-amygdala pathway in non-human primates. This pathway would be involved in unconscious emotion perception responsible for fast behavioral reactions to threatening stimuli (Jones and Burton, 1976; Liddell et al., 2005; Tamietto et al., 2012; Elorette et al., 2018).

Using functional magnetic resonance imaging (fMRI), Kastner et al. (2004) found regions in the mediodorsal right and left pulvinar activated by bilaterally presented flickering checkerboard stimuli, when subjects attended to the stimulus. These regions strongly modulated by visuospatial attention may be homologous to area Pdm of the macaque pulvinar (Petersen et al., 1985). Cotton and Smith (2007) reported a distinct region characterized by visual sensitivity, both in the presence and in the absence of attention, in the inferior pulvinar that responds preferentially to contralateral visual stimulation.

Recent works using high-resolution fMRI investigated the functional organization of the human pulvinar and its connectivity with the cortex (Arcaro et al., 2015, 2018). They identified two visual field maps within the lateral half of the ventral pulvinar. The more medial region, vPul1, appears to correspond to the P1, and the more lateral region, vPul2, appears to correspond to P2 in monkeys (Gattass et al., 1978a; Bender, 1981). Additional representations of the visual space were identified within ventral medial and dorsal lateral regions of the pulvinar, which may correspond to regions P3 and P4 of the macaque, respectively (Gattass et al., 2014). Functional connectivity analyses revealed two pulvino-cortical networks. The dorsal pulvinar was functionally linked to frontal, parietal, and cingulate areas involved in attentional control, tool processing, and the default mode network. The ventral pulvinar was functionally linked to occipital and temporal areas involved in form, object, and scene recognition, consistent with anatomical studies in monkeys (Baleydier and Mauguiere, 1985; Baizer et al., 1993; Webster et al., 1993; Soares et al., 2001; Arcaro et al., 2018).

Analysis of coactivation profiles reported in functional neuroimaging studies (Barron et al., 2015; Guedj and Vuilleumier, 2020), identified five clusters in the pulvinar: dorsomedial, lateral, anterior, inferior and ventromedial. Each cluster representing a region with distinct functional coactivation was compared to human cytoarchitectural divisions reported by Morel et al. (1997). The PuI was almost completely contained within the inferior cluster. The PuA was almost completely contained within the anterior cluster. PuL was split between the inferior and lateral clusters and the PuM was distributed among all five clusters nearly equally.

The meta-analytic connectivity modeling method used by Barron et al. (2015) showed action-specific regions of coactivation with the pulvinar in somatosensory regions, basal ganglia, and cerebellum. Cognition-specific regions of coactivation were seen in the middle and medial frontal gyrus, anterior cingulate, insula, superior and inferior temporal lobe, occipital visual areas, and parahippocampus. Emotion specific regions of coactivation were seen in the amygdala, lentiform nuclei, and lingual gyrus. Perception-specific regions included coactivations in the medial and middle frontal gyrus, anterior cingulate, and caudate head.

In a study using track-weighted dynamic functional connectivity (tw-dFC), a technique which combines structural and dynamic functional connectivity, Basile et al. (2024) investigated the structural and functional organization of the human pulvinar complex. They revealed four topographically organized connectivity clusters that, despite delimiting a small number of clusters, show some similarity to the previous parcellations based on functional connectivity (Barron et al., 2015; Guedj and Vuilleumier, 2020). The dorsolateral cluster occupies the most posterior portion of the dorsal pulvinar; the ventral posterior cluster occupies the most caudal portion of the pulvinar; the dorsomedial cluster is situated medially and anteriorly to the dorsolateral cluster, and the ventral anterior cluster is located in the most medial portion of the ventral pulvinar extending toward the dorsolateral boundaries. Each of these clusters showed functional coupling to specific, widespread cortico-subcortical white matter brain networks. A quantitative analysis of the degree of overlap between tw-dFC driven pulvinar parcellation and the atlas-based subdivision showed that PuI was split at 50% of its volume between the posterior and anterior cluster; PuL was mostly occupied by the anterior cluster and, to a lesser extent, by the dorsolateral cluster. The anterior pulvinar (PuA) showed pronounced overlap with the anterior cluster with lesser correspondence to the dorsomedial and dorsolateral cluster. PuM was almost equally subdivided between all four clusters, with slight predominance for the dorsolateral and posterior cluster.

The cluster-specific connectivity patterns also showed some similarities between these fMRI-based studies (Barron et al., 2015; Guedj and Vuilleumier, 2020; Basile et al., 2024). The dorsomedial cluster displayed a similar pattern of connectivity to the cingulum, precuneus, inferior parietal, and prefrontal regions. The anterior cluster described by Basile et al. (2024) was found to be connected to both sensorimotor region, insula, parietal and occipital regions, such as the anterior and ventromedial clusters described by Guedj and Vuilleumier (2020). The posterior cluster described by Basile et al. (2024) was connected to both early and integrative visual regions of the temporal and occipital lobe, as well as to medial prefrontal cortex, similarly to the inferior cluster described by Guedj and Vuilleumier (2020), and the dorsolateral cluster described by Basile et al. (2024) was connected to prefrontal and parietal regions, as for the lateral cluster described by Guedj and Vuilleumier (2020).

This functional topography observed in the human pulvinar complex may be mediated by distinct anatomical channels, that include the anterior thalamic radiation for the dorsomedial pulvinar, the medial parietal bundle of the superior thalamic radiation for the dorsolateral cluster, the temporal bundle of the posterior thalamic peduncle and the optic radiations for the posterior cluster, and the central part of superior thalamic peduncle and the temporal inferior thalamic peduncle for the anterior cluster (Serra et al., 2017; Basile et al., 2024).

Maldonado et al. (2024) investigated the pulvino-temporal connectivity using postoperative diffusion MRI, virtual dissection and post-mortem fiber dissection. They demonstrated the presence of four fundamental fiber contingents which leave the pulvinar destined for the temporal lobe. Each of those components might correspond to a different level of information processing involved in the lexical retrieval process of picture naming. The authors also demonstrated that stimulation of pulvino-temporal connections in the temporal isthmus causes anomia, establishing their causal role in picture naming.

Basile et al. (2025) investigated the organization of the human pulvinar by applying diffusion embedding to tractography, functional connectivity, and receptor coexpression. They identified multiple gradients of structural connections, functional coactivation, and molecular binding patterns. These results converge on a shared representation along the dorso-ventral and medio-lateral axes of the pulvinar, aligning with connectivity transitions from lower-level to higher-order cortical regions. Additionally, it is consistent with the model of the pulvinar-cortical connectivity proposed for non-human primates (Shipp, 2003; Froesel et al., 2024). These findings reinforce the role of the pulvinar in mediating information integration and communication across brain networks at multiple levels of brain organization.

## 5 Clinical implications

Several studies have pointed to a role of the pulvinar in coordinating attentional modulation on visual processing (Chalupa et al., 1976; Gattass et al., 1978b; LaBerge and Buchsbaum, 1990; Robinson and Petersen, 1992; Shipp, 2003). Electrophysiological studies in monkeys have shown enhanced responses in pulvinar neurons during attention tasks (Petersen et al., 1987; Saalmann et al., 2012; Zhou et al., 2016; Fiebelkorn et al., 2019). Monkeys and patients with pulvinar lesions show deficits in filtering out or ignoring irrelevant stimuli that occur at locations other than the one to which they are attending (Petersen et al., 1985, 1987; Desimone et al., 1990). Pulvinar damage can also produce a contralesional deficit in response competition, when target location was not known and ipsilesional stimuli compete for response (Danziger et al., 2004). The role of the pulvinar in suppressing distractors while attending to behaviorally relevant objects is supported by fMRI experiments in healthy humans (Fischer and Whitney, 2012).

Pulvinar-cortical communication depends on the specific characteristics of pulvinar lesion. A patient with more anterior pulvinar damage shows strong spatial attention deficits, despite no deficit in object-based attention. On the other hand, a patient with posterior pulvinar damage shows clear object-based attention deficits but no issues with spatial attention (Arend et al., 2008). Snow et al. (2009) examined the contribution of the human pulvinar to goal-directed selection of visual targets. To this aim, they investigated patients with lesions in the topographically organized ventral pulvinar, providing evidence that the pulvinar plays a role in modulating physical saliency in attentional selection.

Wilke et al. (2010) found that dorsal pulvinar inactivation in the macaque monkey resulted in a spatial neglect syndrome accompanied by visuomotor deficits, including optic ataxia, during visually guided limb movements. Pulvinar lesions are also associated with spatial neglect in humans. However, in some patients, hemispatial neglect after pulvinar lesions is resolved within a few weeks or months (Karnath et al., 2002). Patients examined many months after their initial trauma showed no clinical signs of hemispatial neglect (Danziger et al., 2004). In a study of visuomotor performance, a patient with a lesion centered on the medial portion of the dorsal pulvinar exhibited reach and grasp difficulties, but he did not show any spatial choice bias or perceptual deficit (Wilke et al., 2018).

Olfactory function was tested in patients with unilateral focal lesions involving the pulvinar, the mediodorsal (MD) or the lateral-compartment subregions of the thalamus. Thalamic lesions did not significantly influence olfactory detection but impaired olfactory identification, while lesions on the right side altered olfactory hedonics by reducing the pleasantness of pleasant odors. Pulvinar damage was associated with worse olfactory performance (Sela et al., 2009). Accordingly, Gattass et al. (1978b) showed neuronal response to olfactory stimuli in the PL of the capuchin monkey and Homman-Ludiye and Bourne (2019) provided evidence that the olfactory piriform cortex and the auditory cortex are interconnected through the PM in the marmoset. They thereby propose that PM participates in olfactory perception in primates by synchronizing distinct cortical areas and optimizing multimodal information transmission.

Thalamus pathology involving the pulvinar has been recognized as an important contributor to cognitive decline and behavioral symptoms in Lewy body dementia (LBD) and Parkinson’s disease (PD). In these patients, visual hallucinations, delusions, and functional neurological manifestations become more frequent with the progression of the disease. These diseases are characterized by alfa-synuclein deposition in the form of Lewy bodies in neuronal perikarya and Lewy neurites in cellular processes (Braak et al., 2003; Biesbroek et al., 2024). Erskine et al. (2017) investigated the subregions of the pulvinar in postmortem tissue taken from LBD patients who had experienced visual hallucinations during life. They found a significant loss in the number of neurons in the lateral but not in the anteromedial pulvinar, while Lewy body pathology was most marked in PM. Combined MRI and DTI study of the visual thalamus in LBD patients found micro-structural alteration in the regions projecting from pulvinar to the parietal and occipital cortices and demonstrated that the degree of degeneration in the pulvinar was associate with visual hallucination frequency and severity (Delli Pizzi et al., 2014). Furthermore, an MRI study found differences in the left medial pulvinar between LBD patients and neurotypical controls that were associated with the development of executive dysfunction in LBD (Tak et al., 2020).

Recently, Ferrer-Gallardo et al. (2025) used a high-resolution *ex vivo* MRI technique to investigate volume changes in thalamic nuclei associated with cognitive and motor deficits in Parkinson’s disease. They found that cognitive alterations were linked to volume changes mainly in the anteroventral and ventral posterolateral nuclei. Motor impairment was associated with volume lateralization asymmetries in the centromedian, mediodorsal medial, pulvinar anterior, and pulvinar medial nuclei. The lateralization asymmetries in the volumes of PuM and PuA found in association with motor deficits could be linked to the pulvinar role in visual motor planning, typically attributed to the pulvinar projections to the frontal and parietal eye fields (Wilke et al., 2010; Dominguez-Vargas et al., 2017; Ferrer-Gallardo et al., 2025). Matsuura et al. (2019, 2023) reported on the relationship between low pulvinar nuclei intensity in susceptibility-weighted imaging and the appearance of visual hallucinations and cognitive decline. They studied the appearance of visual hallucinations after DBS surgery, a beneficial therapy for motor fluctuation in patients with advanced PD. This study revealed that the hypointensity susceptibility mapping in the pulvinar and putamen predicts cognitive deterioration as well as visual hallucinations after deep brain stimulation in Parkinson’s disease patients.

Neuroimaging studies investigated volumetric change and functional connectivity of the pulvinar in patients with posterior cortical atrophy (PCA), considered as a visual variant of Alzheimer’s disease. PCA patients presented deficits in cognitive and visual functions including visuospatial processing, visual perception, episodic memory, and naming. Pulvinar atrophy as well as impaired functional connectivity between the pulvinar and the parietal and temporal lobes were noted in PCA patients (Fredericks et al., 2019; Wang J. et al., 2024).

Pulvinar damage is also related to neurodevelopmental disorders like autism spectrum disorder (ASD), schizophrenia and attention deficit and hyperactivity disorder (ADHD). Postmortem neuropathological and MRI studies in schizophrenia patients revealed that the medial pulvinar (PM) and (to a lesser extent) the lateral pulvinar (PL) exhibit reduced volumes (Gilbert et al.,2001; Byne et al., 2001, 2002; Danos et al., 2003; Highley et al., 2003; Huang et al., 2024). Some of these studies found a certain degree of lateralization with smaller volumes in the right posterior thalamus (Gilbert et al., 2001; Highley et al., 2003). Functional connectivity studies in patients with schizophrenia revealed reduced connectivity between PM and the temporal, parietal and frontal cortices (Cobia et al., 2017; Penner et al., 2018; Yamamoto et al., 2018). These findings suggest that the thalamus plays a crucial role in disrupting distributed brain circuits, impacting cognition and symptoms over time.

Autism spectrum disorder (ASD), a condition characterized by impairments in social interactions and communication, memory processing as well as repetitive and stereotyped behavior, involves atypical development across multiple subcortical brain regions. Structural MRI, fMRI, and DTI studies have observed volumetric alterations in the thalamus, amygdala, basal ganglia and hippocampus (Mosconi et al., 2009; Nair et al., 2013; Richards et al., 2020; Christensen et al., 2025). Schuetze et al. (2016) analyzed T1-weighted anatomical images from individuals with ASD and observed a specific and persistent increase in the surface area of medial and posterior thalamus regions corresponding to the pulvinar and mediodorsal nucleus. The increase in surface area in left medial thalamus was correlated with the severity of the disorder. Studies on thalamocortical functional connectivity in individuals with ASD found increased connectivity of the medial region of the pulvinar with the prefrontal and temporal cortices (Woodward et al., 2017; Baran et al., 2023).

Recent studies have proposed epilepsy as a network disease, characterized by the activation of interconnected cortical as well as subcortical regions (Spencer, 2002; Blumenfeld, 2014). Imaging studies (MRI) have provided relevant information for the characterization of macroscopic network abnormalities in the epileptic brain (Szabo et al., 2005; Raghavendra et al., 2007; Nakae et al., 2016; Sarria-Estrada et al., 2022). MRI abnormalities were seen more frequently in patients with prolonged status epilepticus (SE) and tended to be more prevalent among patients with cortex and pulvinar lesions than among patients with cortex lesions alone (Nakae et al., 2016). These patients also presented lateralized periodic discharges (LPDs) on the EEG, attributed to a disconnection between the cortex and subcortical structures, which was strongly associated with SE-related MRI abnormalities, particularly in the hippocampal and pulvinar regions. Specific alterations in the hippocampus and pulvinar were related to the temporal origin of the SE (Sarria-Estrada et al., 2022).

Researchers have shown that an interruption or a modification of the network activity by electrical stimulation may alter seizure expression or its occurrence. Deep brain stimulation (DBS) has emerged as a promising neuromodulation technique for drug-resistant epilepsy. Stimulation has been directed at subcortical structures such as the anterior and centromedian nuclei, the hippocampus, and more recently the pulvinar (Vetkas et al., 2022; Wang R. et al., 2024; McGinn et al., 2024; Gregg et al., 2025; Feys et al., 2025). The involvement of the medial pulvinar in the pathophysiology of temporal lobe epilepsy has been investigated using SEEG during spontaneous temporal lobe epilepsy. Epileptic changes in PM activity were frequently observed during seizures, implying PM in epilepsy transmission (Rosenberg et al., 2006; Soulier et al., 2023). Analysis of ictal MRI to detect subcortical changes during SE frequently showed thalamic diffusion restrictions in PM. Thalamic DWI-abnormalities in PM was strongly associated with temporal status epilepticus, but PM was less frequently involved in parietal status epilepticus and only rarely in frontal status epilepticus (Capecchi et al., 2020). Electrical stimulation of PM reduces the severity of seizures originating in the medial temporal lobe and posterior quadrant (Filipescu et al., 2019; Burdette et al., 2021; Vakilna et al., 2023; Wang R. et al., 2024).

High-frequency PM stimulation has been shown to reduce seizure severity and improve awareness, potentially by disrupting excessive pathological thalamocortical synchronization (Acerbo et al., 2025; Bratu et al., 2025; Feys et al., 2025). Acerbo et al. (2025) investigated the impact of different stimulation frequencies on brain functional connectivity (FC) in patients undergoing presurgical evaluation implanted with stereoelectroencephalographic (SEEG) electrodes in PM. They observed a decrease in FC with high-frequency stimulation (>90 Hz, up to 150 Hz) that extended beyond the epileptogenic zone, significantly affecting all brain lobes, particularly the parietal, insular, and subcortical regions, contrasting with an increase in FC in the 20–80 Hz range. These results highlight the effects of PM stimulation on FC patterns, supporting the role of high-frequency stimulation as a promising strategy for reducing interictal FC in epilepsy patients. Bratu et al. (2025) investigated the effects of PM stimulation on signal complexity and its relationship to ictal awareness using permutation entropy in SEEG recordings. The results showed that PM stimulation attenuated entropy reductions during seizures, suggesting a preservation of neural complexity, with potential implications for preserving both consciousness and cognitive function in epilepsy.

Bilderbeek et al. (2025) delivered electrical stimulation to different pulvinar subregions and measured the resulting evoked potentials in patients with drug-resistant epilepsy to verify whether different lateral temporal and posterior quadrant brain areas are preferentially influenced by different pulvinar subregions. Stimulation of PL elicited evoked potentials in striate and extrastriate visual areas that diminished as the stimulation shifted toward PM. Stimulation of the ventral portion of PM evoked lateral temporal evoked potentials, which diminished with PL stimulation. Stimulation of the dorsomedial pulvinar evoked parietal responses with limited striate/extrastriate and lateral temporal responses. These results corroborate previous tracer studies in monkeys and human DTI and fMRI studies that found similar structural and functional connectivity between the pulvinar and selected cortical regions (Kaas and Lyon, 2007; Arcaro et al., 2018; Cortes et al., 2024).

## 6 Conclusion

The pulvinar is a higher-order thalamic nucleus that exhibits a conserved organizational framework across primate species, despite notable differences in the size of its subdivisions and in the complexity of their individual connectivity patterns. In humans, for example, the medial pulvinar is markedly enlarged, paralleling the expansion of the cortical areas with which it is interconnected. Interest in pulvinar research has grown substantially in recent years, driven by the recognition that the pulvinar participates in multiple circuits performing diverse underlying functions. That is particularly true for pulvino-cortical interactions involved in attentional networks, where it appears to act not only as a hub for the coordination of information processing, but also as a modulator of cortical excitability and a synchronizer across interconnected regions.

Human neuroimaging studies (fMRI, DWI) corroborate connectivity and electrophysiological findings in non-human primates and thereby hold great promise as these technologies continue to advance. High-resolution imaging may be able to better delineate the functional heterogeneity of pulvinar subnuclei, which may enable targeted interventions with greater specificity. Complementary evidence from inactivation and lesion studies of different pulvinar regions, together with stimulation and connectivity approaches further highlights the pulvinar’s role in neurological disorders. Nevertheless, the fine anatomical organization of pulvinar subdivisions and their connections with diverse cortical regions still requires more precise characterization. Therefore, we must continue to explore the effects of activating or silencing specific components of these circuits to fully elucidate their physiological roles and pathological involvement. Advances in multi-electrode recordings, optogenetics, and connectomic techniques are expected to significantly enhance this understanding in both animal models and humans. Moreover, the integration of electrophysiological recordings with stimulation paradigms holds promise for refining therapeutic strategies in epilepsy and psychiatric disorders. New studies that combine high resolution multimodal mapping in humans (7T fMRI, diffusion MRI, MEG/EEG) with computational models that translate neural dynamics into observable measures are also important to generate testable predictions. Collectively, these coordinated efforts will point toward new avenues for diagnosis and therapeutic interventions.
